# Mapping QTLs for grain yield components in wheat under heat stress

**DOI:** 10.1371/journal.pone.0189594

**Published:** 2017-12-19

**Authors:** Nabin Bhusal, Ashok Kumar Sarial, Pradeep Sharma, Sindhu Sareen

**Affiliations:** 1 Department of Genetics and Plant Breeding, CCS Haryana Agricultural University, Hisar, Haryana, India; 2 Department of Genetics and Plant Breeding, CCS Haryana Agricultural University, College of Agriculture Campus Kaul, Kaithal, Haryana, India; 3 ICAR-Indian Institute of Wheat and Barley Research, Aggarsain Marg, Karnal, Haryana, India; Institute of Genetics and Developmental Biology Chinese Academy of Sciences, CHINA

## Abstract

The current perspective of increasing global temperature makes heat stress as a major threat to wheat production worldwide. In order to identify quantitative trait loci (QTLs) associated with heat tolerance, 251 recombinant inbred lines (RILs) derived from a cross between HD2808 (heat tolerant) and HUW510 (heat susceptible) were evaluated under timely sown (normal) and late sown (heat stress) conditions for two consecutive crop seasons; 2013–14 and 2014–15. Grain yield (GY) and its components namely, grain weight/spike (GWS), grain number/spike (GNS), thousand grain weight (TGW), grain filling rate (GFR) and grain filling duration (GFD) were recorded for both conditions and years. The data collected for both timely and late sown conditions and heat susceptibility index (HSI) of these traits were used as phenotypic data for QTL identification. The frequency distribution of HSI for all the studied traits was continuous during both the years and also included transgressive segregants. Composite interval mapping identified total 24 QTLs *viz*., 9 (timely sown traits), 6 (late sown traits) and 9 (HSI of traits) mapped on linkage groups 2A, 2B, and 6D during both the crop seasons 2013–14 and 2014–15. The QTLs were detected for GWS (6), GNS (6), GFR (4), TGW (3), GY (3) and GFD (2). The LOD score of identified QTLs varied from 3.03 (*Qtgns*.*iiwbr-6D*) to 21.01 (*Qhsitgw*.*iiwbr-2A*) during 2014–15, explaining 11.2 and 30.6% phenotypic variance, respectively. Maximum no of QTLs were detected in chromosome 2A followed by 6D and 2B. All the QTL detected under late sown and HSI traits were identified on chromosome 2A except for QTLs associated with GFD. Fifteen out of 17 QTL detected on chromosome 2A were clustered within the marker interval between *gwm448* and *wmc296* and showed tight linkage with *gwm122* and these were localized in 49–52 cM region of Somers consensus map of chromosome 2A i.e. within 18–59.56 cM region of chromosome 2A where no QTL related to heat stress were reported earlier. Besides, three consistent QTLs, *Qgws*.*iiwbr-2A*, *Qgns*.*iiwbr-2A* and *Qgns*.*iiwbr-2A* were also detected in all the environments in this region. The nearest QTL detected in earlier studies, *QFv/Fm*.*cgb-2A* was approximately 6cM below the presently identified QTLs region, respectively Additionally, QTLs for physiological and phenological traits and plant height under late sown and HSI of these traits were also detected on chromosome 2A. QTL for HSI of plant height and physiological maturity were located in the same genomic region of chromosome 2Awhereas QTLs for physiological and phonological traits under late sown were located 8cM and 33.5 cM below the genomic location associated with grain traits, respectively in consensus map of Somers. This QTL hot-spot region with consistent QTLs could be used to improve heat tolerance after validation.

## Introduction

Since 1980s global wheat productivity has reduced by as much as 5% due to increase in temperature roughly by 0.13°C per decade since 1950[1–2]. IPCC predicted in 2012 [3] that the world daily maximum temperature will rise approximately 1.3°C by middle and 2–5°C by the end of 21^st^ century. At the same time, South Asia will face an increase of 1.54°C in maximum and 1.08°C in minimum temperature during rabi (wheat) season by 2020. In India, central and peninsular zone experiences heat stress throughout crop season whereas, northwestern plain zone faces terminal heat stress due to delayed sowing [4–5]. Approximately 13.5 million ha of wheat growing area is affected by heat stress [6].

The optimum temperature for wheat crop during the post-anthesis period is 22-25ºC, beyond that it feels the heat; causing irreversible damage by high temperature [7]. It has been reported that each °C rise in temperature above cardinal causes reduction in grain filling duration by 2.8 days [8], grain numbers by 4% [9], gain weight by 5% [10] and grain yield by 3–4% [11]. In 2004, the country suffered a yield loss of 4.6 Mt due to the sudden increase in temperature during February [12].

Strategies that improve tolerance to heat stress would be beneficial for wheat production. Selecting traditionally grown heat-tolerant cultivars in warmer regions may serve as one of the strategies to reduce heat stress-related losses because of global warming [13–14]. The development of genotypes with potentially high yield under heat stress conditions has led to modest genetic gains [15]. The limited understanding of this complex quantitatively inherited phenomenon controlled by numerous interacting QTLs/genes, its genetic and molecular mechanisms of whole plant adaptation has restricted the major breakthrough in breeding for heat tolerance. Detection of QTL associated with various complex traits allows the detection of chromosomal segments controlling these traits would be beneficial in the breeding program. However, expressions of these interacting QTLs are modified by the environment [16–17]. QTLs for various traits under heat stress have been previously detected on all the 21 chromosomes; maximum on 3B (33) followed by 2D (30), 5A (29), 7D (19), 7A (18), 1B (17), 2B (16), 4A (15), 5B (15), 2A (14), 6A (13), 1A (12), 4B (12), 4D (12), 7B (12) 6B (9), 1D (7), 3A (4), 5D (4), 6D (4) and 3D (2).The present study further steps to identify QTLs for grain yield and its’ component traits which can be utilized in marker-assisted selection and breeding after validation.

## Materials and methods

Director, ICAR-IIWBR (earlier known as Directorate of Wheat Research), approved the work plan of the project CRSCDWRSIL201001400105 (DWR/RP/10-5.6) during Institute Research Council meeting.

### Plant material

A mapping population of 397 recombinant inbred lines derived from a cross between HD2808 (heat tolerant) and HUW510 (heat susceptible) was used in the study. HD2808 is an advanced breeding line from a cross betweenWH542/DL377-8 developed in 1995 [18]. HUW510 (HD2278/HUW234//DL230-16) was developed in 2002 for timely sown and irrigated conditions. Both these genotypes were evaluated for terminal heat stress tolerance under field and temperature controlled conditions during the crop seasons, 2006-07and 2007–08 at IIWBR, Karnal. On confirmation of their respective tolerance and susceptibility for the grain traits, the crosses were effected during the 2007–08 crop season. The F_1_ and F_2_ were raised in 2008–09 and 2009–10 crop seasons. Subsequently, generations were advanced to the F_6_generation following single seed descent method during the off-season at Dalang Maidan experimental station and main season at IIWBR, Karnal. The F_6_ and F_7_ generations consisting of 397 RILs along with parents were then evaluated during 2011–12 and 2012–13 crop seasons under timely and late sown conditions. Based on heat susceptibility index of RILs, the subset consisting of 251 lines, which represented the whole variation in grain traits for heat tolerance, was selected for further study.

#### Evaluation for heat stress

The experiment was conducted at Indian Institute Wheat and Barley Research, Karnal (29^0^ 43’ N latitude; 76^0^48’ E, longitude, 245 m above mean sea level and soil pH 7.4) during 2013–14 and 2014–15 crop seasons under two planting conditions, timely (mid November) and late sown (mid December). All experiments were conducted under irrigated conditions with same planting approach in both years. The experiment was conducted in a randomized complete block design (RBD) with two replications. The seeds of 251 RILs along with parents were hand sown in the plot. The plot area was 0.69m^2^ (3 rows of 1m length and 0.23m of row spacing). Seed rate was kept at 100 kg ha^-1^. Recommended package of practices for the agro-climatic zone was followed to raise the crop experiment. Tilt was sprayed to protect the crop from disease [19]. Data were recorded for phenological traits and grain yield and its components viz., days to heading (Z55), days to anthesis (Z64-65), days to physiological maturity (Z91–92), grain filling duration (Z64-92),biological yield (BY) and grain yield (GY) (at harvesting). Post-harvest data was recorded for grain weight/ main spike (GWS), grain numbers/main spike (GNS), 1000 grain weight (TGW) and grain filling rate (GFR). GFD was estimated as the difference in days between anthesis and physiological maturity. Five random main spikes were harvested from each plot, hand threshed and grain number/spike and grain weight/spike was estimated. TGW was measured by taking random samples of 500 grains from plot yield and weighed. GY and BY were measured after harvesting plots at maturity. GFR was measured as a ratio between single kernel weight and grain filling duration. Heat susceptibility index (HSI) as the measure of heat tolerance for each trait was calculated using the formula given by Fischer and Maurer [20].

Data were subjected to statistical analysis using CROPSTAT 7.2 http://bbi.irri.org/products) [21]and SAS 9.3 (SAS Institute Inc., Cary, NC) [22] software.

### Genotyping

Genomic DNA from 251 RILs and parents was isolated from 30 days old seedlings following modified CTAB extraction method as given by SaghaiMaroof *et al*. [23]. A consensus map of Somers *et al*. [24] containing 1,235 microsatellite markers was used to select markers. Total 380 microsatellite/simple sequence repeat (SSR) markers were selected for initial screening for parental polymorphism. In chromosome 2A total 40 microsatellites were used for parental screening. PCR reaction was performed following Sharma *et al*. [25].

#### Linkage mapping and QTL analysis

Genotyped data of RILs population was used to generate genetic linkage map using software Join Map 4.0 (Kyazma, B.V.,Netherlands). The Kosambi mapping function was used for conversion of recombination into the genetic distance. QTL analysis was performed using QTL Cartographer v2.5 [26]. Before QTL analysis, phenotypic data of HSI for each trait was transformed by natural logarithm (reflected) method based on skewness (http://www.vassarstats.net/trans1.html). Transformed values of HSI for each trait were initially used to perform single marker analysis to identify significant genetic markers associated with phenotypic traits. The trait settings for CIM were done using model 6, forward and backward stepwise regression with a threshold of P/0.05 to select cofactors, window size 10 and 5cM walking speed along chromosomes. QTLs were verified by LOD scores compared to an empirical genome-wide significant threshold calculated from 1,000 permutations for P/0.05. LOD scores and coefficients of determination were estimated by CIM for each QTL. The LOD value was fixed as≥3.0. To find out the epistatic QTLs we used software QTL-Network [27]. The name of QTLs was designated based on the nomenclature in the catalog for gene symbols for wheat (http://wheat.pw.usda.gov/ggpages/wgc/98/). Map and QTL graphics were drawn using software MapChart v2.1 [28].

## Results

### Heat stress

The mean minimum and maximum temperature under late sown condition were higher from timely sown conditions by 2.7 and 4.2°C (2013–14) and 1.1 and 0.9°C (2014–15), respectively (S1 Fig).

#### Phenotyping of RILs and parents

Analysis of variance based on heat susceptibility index showed significant (p < 0.01) main effects due to genotypes for all traits studied during both the crop seasons 2013–14 and 2014–15 (Table 1). Mean performance of parents and RILs under both the sowing conditions and years along with their HSI is presented in S1 Table. HSI values of various traits in HD2808 was <1 and in HUW510 it was >1 during both the crop seasons (2013–14 and 2014–15). The frequency distribution of HSI for all the studied traits was continuous during both the years (S2 Fig). HSI values of RILs exceeded beyond those of the parents revealing the presence of transgressive segregants. During 2013–14 crop season, 2.8%, 2.8%, 10.4% and 18.7% of RILs had HSI less than the heat tolerant parent, HD2808, for GWS, GNS, TGW, and GY. During 2014–15, 23.9%, 23.0%, 25.1% and 11.5% of RILs had HSI less than the heat tolerant parent HD2808 for these traits respectively. Similarly, during 2013–14 crop season, 1.5%, 11.5%, 14.7% and 3.2% exceeded the heat susceptible parent HUW510 for GWS, GNS, TGW, and GY. During 2014–15 crop season, 5.2%, 5.2%, 8.4% and 15.5% RILs exceeded the susceptible parent for these traits.

**Table 1:** Pooled analysis of variance for HSI of various traits of HD 2808/ HUW 510 RIL population.

Source	DF	HSIGFD	HSIGWS	HSIGNS	HSITGW	HSIBY	HSIGY	HSIGFR
Replications	1	0.0012	0.0002	0.0024	0.250	0.031	0.001	0.283
Genotypes	250	0.053**	0.258**	0.297**	0.444**	0.136**	0.196**	0.370**
Years	1	15.70**	0.090	0.039	1.95**	44.88**	0.588**	5.109**
Genotypes X Replications	250	0.010**	0.023**	0.035	0.108	0.026**	0.021**	0.118
Years X Genotypes	250	0.055**	0.129**	0.063**	0.231**	0.144**	0.108**	0.180**
Residual	502	0.010	0.0258	0.029	0.113	0.025	0.021	0.127
Coefficient of Variation (%)		4.71	6.93	8.30	13.31	8.53	6.77	13.47
Heritability		68.01	83.35	78.86	60.70	67.82	80.39	51.71

** Significant at (P< 0.01) percent LSD and * significant at (P<0.05) percent LSD

HSIGFD, Heat susceptibility index of grain filling duration; HSIBY, Heat susceptibility index of biological yield m^-2^; HSIGWS, Heat susceptibility index of grains weight/main spike; HSIGY, Heat susceptibility index of grain yield m^-2^; HSIGNS, Heat susceptibility index of grain number/ main spike; HSITGW, Heat susceptibility index of 1000 grains weight and HSIGFR, Heat susceptibility index of grain filling rate.

#### QTL mapping

Three hundred and eighty SSR primers were screened with parents HD2808 and HUW510 to detect polymorphism. Of these, 14.2% markers revealed parental polymorphism; covering A genome (46.2%), B genome (28.8%) and D genome (25.0%). In chromosome 2A total 40 microsatellites were used for parental screening of these, 35% showed polymorphism. The 52 polymorphic markers were used to construct the linkage map. The linkage group of 2A covered 187.2 cM while, remaining linkage groups representing chromosomes 1A, 2B, 2D, 3A, 5B, 6D, and 7A spanned 671.4cM.

**Fig 1:**
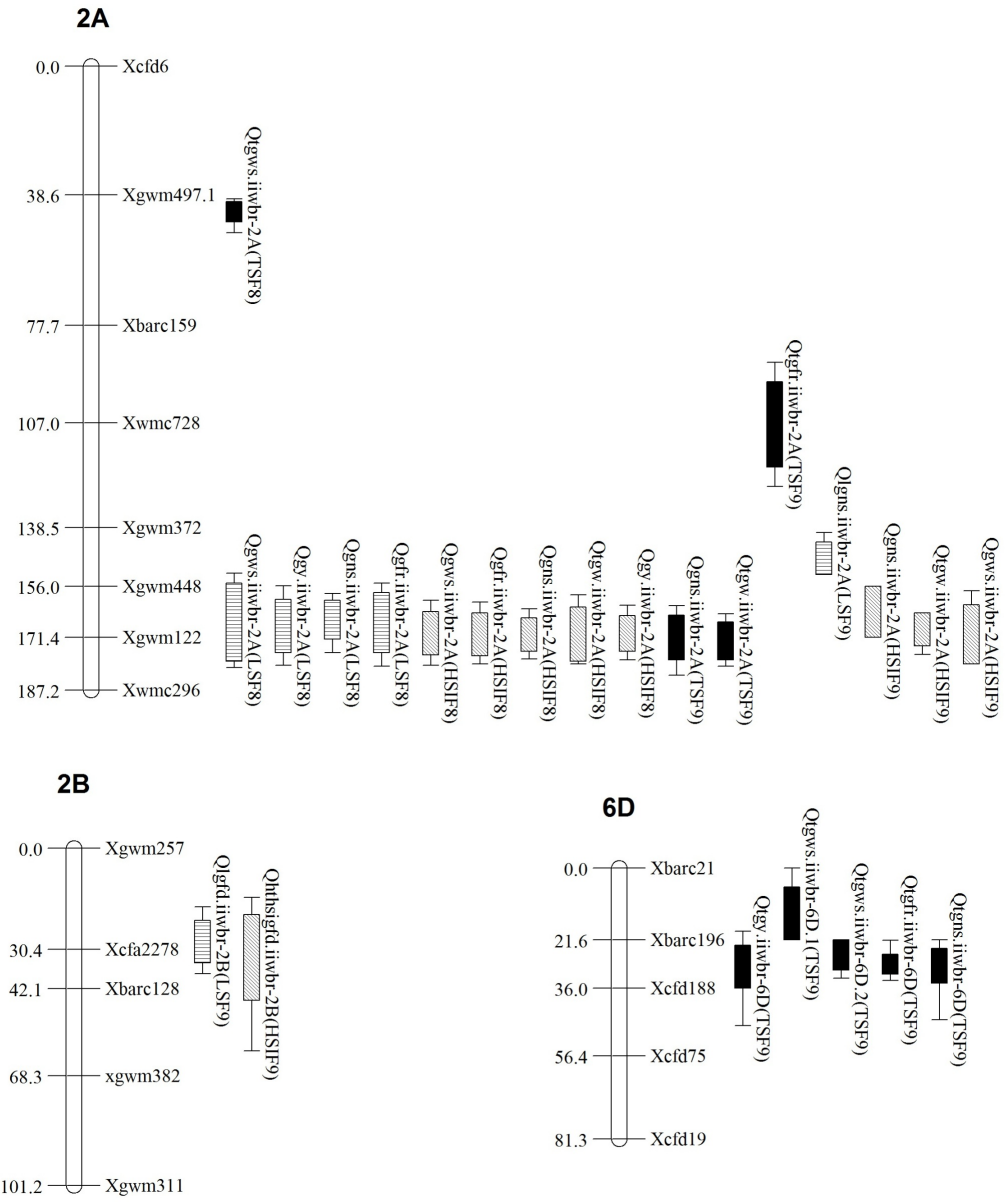
QTLs identified during 2013–14 and 2014–15 crop seasons using HD2808/ HUW 510 RIL population.

Composite interval mapping identified total 24 QTLs *viz*., 9 (timely sown traits), 6 (late sown traits) and 9 (HSI of traits) mapped on linkage groups 2A, 2B, and 6D during both the crop seasons 2013–14 and 2014–15 (Fig 1). The LOD score of identified QTLs varied from 3.03 (*Qtgns*.*iiwbr-6D*) to 21.01 (*Qhsitgw*.*iiwbr-2A*) during 2014–15, explaining 11.2 and 30.6% phenotypic variance, respectively. Maximum no of QTLs were detected in chromosome 2A followed by 6D and 2B. The QTLs were detected for GWS (6), GNS (6), GFR (4), TGW (3), GY (3) and GFD (2) (Table 2). The phenotypic variance explained by these identified QTLs ranged from 7.56 to 28.84% for GWS, 7.20 to 23.16% for GNS, 12.13 to 30.63% for TGW, 6.74 to 16.28% for GFR, 9.46 to 16.26% for GY and 5.67 to 15.23% for GFD. All the QTL detected under late sown and HSI traits were identified on chromosome 2A except for QTLs associated with GFD. The GFD under late sown and HSI traits were associated with QTLs *Qlgfd*.*iiwbr-2B* and *Qhsigfd*.*iiwbr-2B* on chromosome 2B during crop season 2014–15 explaining 5.67 and 23.16% PV, respectively. The QTLs associated with GWS, GNS and TGW i.e. *Qgws*.*iiwbr-2A*, *Qgns*.*iiwbr-2A* and *Qtgw*.*iiwbr-2A* were common and appeared in most of the environments and HSI of these traits on chromosome 2A, explained 7.56 to 19.86%, 7.20 to 20.04% and 12.13 to 30.63% phenotypic variance of these traits, respectively. These QTLs were co-localized with the QTL associated with GY (*Qgy*.*iiwbr-2A*) and GFR (*Qgfr*.*iiwbr-2A*) detected under late sown and HSI traits during 2013–14 crop season explaining 12.1 and 12.8% phenotypic variance, respectively. Under late sown condition during 2014–15 crop seasons, QTL associated GNS (*Qlgns*.*iiwbr-2A*) was detected on chromosome 2A explained 23.16% PV. Under timely sown conditions, QTLs were detected for GWS (*Qtgws*.*iiwbr-6D*.*1* and *Qtgws*.*iiwbr-6D*.*2*), GNS (*Qtgns*.*iiwbr-6D*), GFR (Qtgfr.iiwbr-6D) and GY (*Qtgy*.*iiwbr-6D*) on chromosome 6D. These QTLs explained 13.24 and 8.92%, 11.22%, 16.28% and 16.16% phenotypic variance.

**Table 2:** Effects of QTLs identified using WINQTL Cartographer following method composite interval mapping (CIM) during 2013–14 and 2014–15 in HD 2808/ HUW 510 RIL population.

Trait	Year	QTLs Name	Chromosome	Marker	Additive Effect	Position	Marker Distance	LOD Score	R^2^	Positive allele
**TS_GWS**	2013–14	*Qtgws*.*iiwbr-2A*	**2A**	*Gwm497*.*1*	-0.398	41.61	3.01	4.73	28.85	HUW510
	2014–15	*Qtgws*.*iiwbr-6D*.*1*	6D	*Barc21*	-0.188	15.01	15.01	3.27	13.24	HUW510
		*Qtgws*.*iiwbr-6D*.*2*	6D	*Barc196*	-0.171	24.61	3.03	3.24	8.92	HUW510
**LS_GWS**	2013–14	*Qgws*.*iiwbr-2A*	**2A**	*Gwm122*	0.105	171.41	0.01	7.56	13.11	HD2808
**HSI_GWS**	2013–14	*Qgws*.*iiwbr-2A*	**2A**	*Gwm122*	-0.161	171.41	0.01	12.17	19.86	HD2808
	2014–15	*Qgws*.*iiwbr-2A*	**2A**	*Gwm448*	-0.088	170.01	1.4	4.45	7.56	HD2808
**TS_GNS**	2014–15	*Qgns*.*iiwbr-2A*	**2A**	*Gwm448*	-1.901	171.01	15.11	3.89	7.20	HUW510
		*Qtgns*.*iiwbr-6D*	6D	*Barc196*	-2.82	27.61	6.03	3.03	11.22	HUW510
**LS_GNS**	2013–14	*Qgns*.*iiwbr-2A*	**2A**	*Gwm448*	2.466	165.01	9.11	7.72	17.02	HD2808
	2014–15	*Qlgns*.*iiwbr-2A*	**2A**	*Gwm372*	2.792	149.01	10.51	9.67	23.16	HD2808
**HSI_GNS**	2013–14	*Qgns*.*iiwbr-2A*	**2A**	*Gwm122*	-0.137	171.41	0.01	10.36	15.87	HD2808
	2014–15	*Qgns*.*iiwbr-2A*	**2A**	*Gwm448*	-0.138	166.51	4.89	10.16	20.04	HD2808
**TS_TGW**	2014–15	*Qtgw*.*iiwbr-2A*	**2A**	*Gwm122*	-2.092	174.41	3.01	14.21	23.69	HUW510
**HSI_TGW**	2013–14	*Qtgw*.*iiwbr-2A*	**2A**	*Gwm122*	-0.131	168.01	3.39	5.65	12.13	HD2808
	2014–15	*Qtgw*.*iiwbr-2A*	**2A**	*Gwm122*	-0.269	171.41	0.01	21.01	30.63	HD2808
**LS_GFD**	2014–15	*Qlgfd*.*iiwbr-2B*	2B	*Cfa2278*	0.752	30.41	0.01	3.12	5.67	HD2808
**HSI_GFD**	2014–15	*Qhthsigfd*.*iiwbr-2B*	2B	*Gwm257*	-0.128	28.01	28.01	3.74	15.23	HD2808
**TS_GFR**	2014–15	*Qtgfr*.*iiwbr-2A*	**2A**	*Wmc728*	-0.73	107.01	0.01	3.71	6.74	HUW510
		*Qtgfr*.*iiwbr-6D*	6D	*Barc196*	-1.53	27.61	26.03	4.46	16.28	HUW510
**LS_GFR**	2013–14	*Qgfr*.*iiwbr-2A*	**2A**	*Gwm448*	0.895	169.51	13.61	4.40	8.74	HD2808
**HSI_GFR**	2013–14	*Qgfr*.*iiwbr-2A*	**2A**	*Gwm122*	-0.142	171.41	0.01	7.38	12.82	HD2808
**TS_GY**	2014–15	*Qtgy*.*iiwbr-6D*	6D	*Barc196*	-0.058	27.61	6.03	3.64	16.26	HUW510
**LS_GY**	2013–14	*Qgy*.*iiwbr-2A*	**2A**	*Gwm448*	0.032	169.51	13.61	4.76	9.46	HD2808
**HSI_GY**	2013–14	*Qgy*.*iiwbr-2A*	**2A**	*Gwm122*	-0.107	171.41	0.01	7.31	12.15	HD2808

TS:Timely sown, LS:Late sown, HSI:Heat susceptibility index

## Discussion

### Heat stress

The mean minimum and maximum temperatures, one week after heading under late sown (stress) condition were higher than the timely sown (normal) condition by 3.4 and 2.5°C during 2013 14 and by 4.6 and 1.2°C during 2014–15 crop seasons. At grain filling stage the mean minimum and maximum temperature under late sown conditions were higher than timely sown conditions by 2.8 and 5.1°C in 2013–14 and 1.6 and 2.7°C in 2014–15 crop seasons. Increase in temperature under late sown crop initiated early anthesis and forced maturity which significantly reduced grain filling duration by 8 to 10 days. The high temperature (>30°C) stress was initiated during the 3rd week after heading in 2013–14 crop season and during the 5^th^ week after heading in the 2014–15 crop season. The temperature recorded over both years in the present investigation showed late sown wheat faced terminal heat stress. However, heat stress during 2013–14 crop season was comparatively more than 2014–15 at post heading stage i.e anthesis and grain filling.

#### Phenotyping

Analysis of variance showed that genotypes were significantly different for HSI of all the traits during both years. As expected, HD2808 had <1.0 and HUW510 had>1.0 HSI for all the traits studied confirming that HD2808 is heat tolerant and HUW510 heat susceptible genotype and the parents were contrasting for response to heat stress for studied traits. High range of HSI for studied traits indicated that genetic component for heat tolerance was well segregated among the RILs in course of their development represented through the normal distribution for studied traits, which showed ample opportunity to detect QTLs associated with heat tolerance. However, RILs also showed transgressive segregation; exceeding both the parents for all studied traits indicated genes with positive and negative effects were dispersed between parents. Rieseberg *et al*. [29] suggested complementary gene action as a primary source of transgression. That may arise during the development of RIL population F_2_ onwards due to accumulation of genes with like effect. The frequency of the transgressive segregants *i*.*e*. RILs exceeded the tolerant parent for GWS, GNS, and TGW were more in 2014–15 than 2013–14 as the former crop season was less heat stressed. Estimates of HSI for each single yield component traits were separately used for mapping.

#### Genotyping

**Fig 2:**
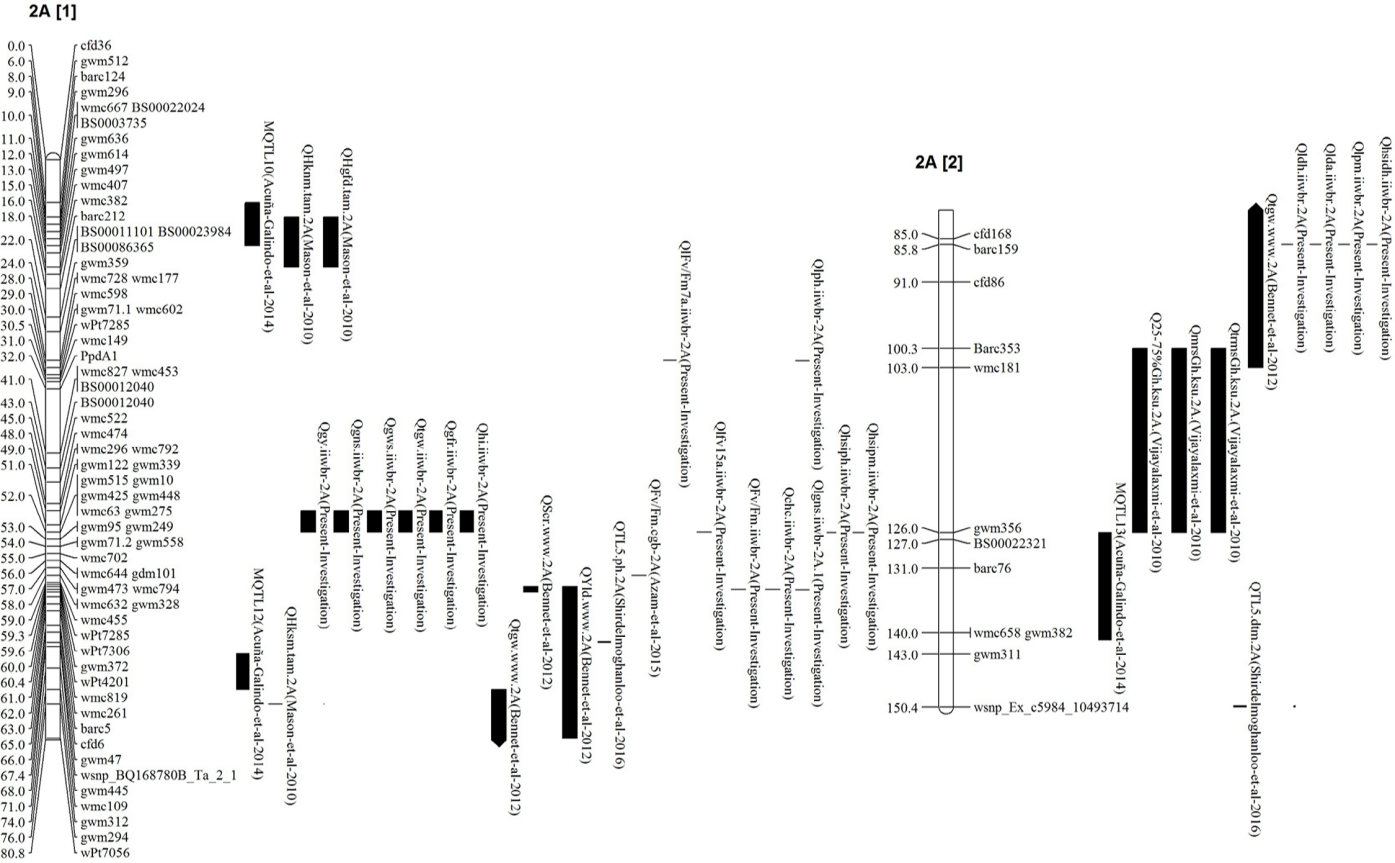
Previously identified QTLs for heat tolerance traits in various studies with respect to QTLs identified in the present investigation on chromosome 2A. Positions of linked markers for the QTL were used as the positions on consensus map of Somers *et al*. [24], Crossa *et al*. [35] and consensus map of Cavanagh *et al*. [36] for SSRs, DArT and SNP markers respectively.

Composite interval mapping revealed genomic regions on chromosomes 2A and 2B were associated with traits under heat stress while the genomic region on 6D was associated with non-stress conditions. QTL *Qgy*.*iiwbr-2A*, detected for grain yield under late sown and HSI on chromosome 2A, was 0.01cM away from linked marker *gwm122*. Acuña-Galindo *et al*.[30] detected MetaQTL (MQTL13) associated with grain yield under heat and drought tolerance on chromosome 2A. Using consensus map of Somers *et al*. [24] as a reference, QTL detected in the present investigation was 75cM away from *MQTL13* indicating that it is novel QTL (Fig 2). Earlier, QTL for HSIGY were reported on chromosome 7B [31], 1D [32], 2D and 5D [33]. Under non-stress conditions, QTL *Qtgy*.*iiwbr-6D* was linked with marker *barc196*. Earlier, QTLs for grain yield under normal condition were reported on chromosome 5B [34], 1A and 7D [32]. Epistatic interaction played a significant role in the expression of traits under heat stress, genomic locations on chromosome 2A, 2D and 3A showed QTL x QTL interaction for HSIGY (Table 3). First interaction was detected on chromosome 2A, *Qhsigy*.*iiwbr-2A*.*1* and *Qhsigy*.*iiwbr-2A*.*2* with marker interval *cfd6-gwm497*.*1* and *gwm122-wmc296* explaining 5.15% phenotypic variance while second epistatic interaction was identified on chromosome 2D and 3A *Qhsigy*.*iiwbr-2D* and *Qhsigy*.*iiwbr-3A* linked with marker interval *gdm6-wmc18* and *barc171-wmc527* explaining 5.04% phenotypic variance.

**Table 3:** Effects of epistatic QTLs under timely (TS), late sown (LS) and HSI for grain yield and component traits using HD2808/HUW 510 RIL population during 2013–14 and 2014–15.

Traits	QTL I	Marker Interval I	Position I	QTL J	Marker Interval J	Position J	additive	R^2^	P value
**During crop season 2013–14**									
**TS_GFD**	*Qtgfd*.*iiwbr-2B*	*cfa 2278-barc 128*	30.4	*Qtgfd*.*iiwbr-6D*	*barc21-barc 196*	0.00	-0.503	5.20	0.000
**TS_GWS**	*Qtgws*.*iiwbr-2A*	*gwm 448-gwm 122*	166.7	*Qtgws*.*iiwbr-2D*	*gdm 6-wmc18*	71.9	0.708	4.81	0.000
**LS_GFD**	*Qlgfd*.*iiwbr-2D*	*gdm 6-wmc 18*	71.2	*Qlgfd*.*iiwbr-7A*	*cfd 20*.*1-gwm 63*	78.3	0.574	4.56	0.005
**HSI_GFR**	*Qhsigfr*.*iiwbr-2A*	*gwm 497*.*1-barc 159*	75.8	*Qhsigfr*.*iiwbr-6D*	*barc21-barc 196*	0.00	-0.093	3.36	0.0005
**During crop season 2014–15**									
**TS_GWS**	*Qtgws*.*iiwbr-2A*	*gwm 372-gwm 448*	151.9	*Qtgws*.*iiwbr-5B*	*wmc 47-barc 4*	0.00	-0.469	16.25	0.00
**TS_GNS**	*Qtgns*.*iiwbr-2A*	*wmc 728-gwm 372*	107.0	*Qtgns*.*iiwbr-2D*	*wmc18-barc 228*	88.3	-2.463	1.77	0.008
**TS_TGW**	*Qttgw*.*iiwbr-2A*	*wmc 728-gwm 372*	118.03	*Qttgw*.*iiwbr-7A*	*cfd 20*.*1-gwm 63*	68.2	-5.345	7.81	0.000
**TS_BY**	*Qtby*.*iiwbr-2B*	*gwm 382-gwm 311*	100.5	*Qtby*.*iiwbr-6D*	*barc 196-cfd 188*	29.6	0.116	7.81	0.000
**LS_BY**	*Qlby*.*iiwbr-2D*	*wmc 453-wmc 111*	0.0	*Qlby*.*iiwbr-5B*	*wmc 47-barc 4*	0.00	-0.051	3.41	0.001
**HSI_TGW**	*Qhsitgw*.*iiwbr-2A*	*gwm 122-wmc 296*	176.1	*Qhsitgw*.*iiwbr-5B*	*wmc 47-barc 4*	13.0	-0.362	1.1	0.01021
**HSI_GY**	*Qhsigy*.*iiwbr-2A*.*1*	*cfd 6-gwm 497*.*1*	25.0	*Qhsigy*.*iiwbr-2A*.*2*	*gwm122-wmc 296*	179.1	-0.702	5.15	0.0003
	*Qhsigy*.*iiwbr-2D*	*gdm 6-wmc 18*	65.8	*Qhsigy*.*iiwbr-3A*	*barc 171-wmc 527*	0.00	-0.112	5.03	0.000
**HSI_GFR**	*Qhsigfr*.*iiwbr-1A*	*gwm 497-wmc 24*	91.1	*Qhsigfr*.*iiwbr-7A*	*cfd 20*.*1-gwm 63*	68.2	-0.246	3.76	0.000
	*Qhsigfr*.*iiwbr-2A*.*1*	*cfd 6-gwm 497*.*1*	32.0	*Qhsigfr*.*iiwbr-2A*.*2*	*gwm 122-wmc 296*	178.9	-0.751	6.26	0.0008
	*Qhsigfr*.*iiwbr-2D*	*barc 228-gwm 382*.*1*	135.4	*Qhsigfr*.*iiwbr-6D*	*barc 21-barc 196*	0.00	0.0756	1.06	0.00376

The major QTL for HSIGFD, *Qhsigfd*.*iiwbr-2B* was 28.01cM away from marker *gwm257*. QTLs mapped on to this region were more prominently associated with heat tolerance for GFD. Major QTLs for HSIGFD had also been reported on this chromosome by Paliwal *et al*. [31]. In addition to that QTLs for HSIGFD were also reported on chromosomes 1D, 2A, 6D [37] and 2D and 7A [32]. The epistatic interactions between *Qlgfd*.*iiwbr-2D* on 2D and *Qlgfd*.*iiwbr-7A* on 7A explained 4.56% phenotypic variance for GFD under stress condition. Earlier, epistatic interaction for grain filling duration under heat stress condition has been reported on chromosome 5B and 7A [32]. Under non-stress conditions, the epistatic interactions between *Qtgfd*.*iiwbr-2B* on 2B and *Qtgfd*.*iiwbr-6D* on 6D explained 5.20% phenotypic variance for GFD.

*Qgws*.*iiwbr-2A*, flanked between marker interval *gwm448* and *wmc296* covering 37cM distances and 0.01cM away from linked maker *gwm122*, appeared for HSIGWS as well as stress environments (S3 Fig). Its appearance in both years indicated its consistency and stability. However; the difference in contribution reflects the effect of environmental factors on the expression of the trait. Mason *et al*. [37] also reported QTL for HSIGWS on chromosome 2A. In addition, QTLs for GWS under non-stress condition were detected on chromosomes 2A and 6D during the crop seasons 2013–14 and 2014–15, respectively. In chromosome 2A detected QTL, *Qtgws*.*iiwbr-2A* under the non-stress condition was 3.01cM away from linked marker *gwm497*.*1* and explained 28.85% phenotypic variance while, under non-stress condition, QTLs *Qtgws*.*iiwbr-6D*.*1* and *Qtgws*.*iiwbr-6D*.*2* were linked with markers *barc21* and *barc196*and explained 13.24 and 8.92% phenotypic variance, respectively. QTL x QTL interactions for this trait was prominent under optimum conditions during the crop season 2014–15 on chromosomes 2A and 5B explaining 16.25% phenotypic variance.

QTL *Qgns*.*iiwbr-2A* for grain number/main spike mapped on chromosome 2A also appeared across the environments (S4 Fig). It co-localizes with *Qgws*.*iiwbr-2A* and explained 7.2 to 20.1% phenotypic variance. However, its contribution under stress conditions and for HSI of the trait was higher indicating that identified QTL was more prominent under heat stress. Another QTL *Qlgns*.*iiwbr-2A* on chromosome 2A was associated with stress conditions and was 10.51cM away from linked marker *gwm372*. QTL associated with GNS were earlier also reported on chromosome 2A [37–38]. In case of normal condition QTL *Qtgns*.*iiwbr-6D*, which was 6.03cM away from linked marker *barc196*, was associated with non-stress conditions. The epistatic interactions for this trait under non-stress conditions on genomic location on chromosome 2A and 2D explained 1.77% phenotypic variance.

*Qtgw*.*iiwbr-2A* appeared across the environments and was flanked between marker interval *gwm448* and *wmc296* covering 37 cM distances and was tightly linked with maker *gwm122* (S5 Fig). The phenotypic variance explained by this QTL ranged from 12.13 to 30.6%. The earlier reports for QTLs associated with TGW under heat stress are on chromosomes 2B, 7B, 7D [31], 2D, 5A [33] and 1D and 6B [32]. QTL x QTL interaction for HSI of this trait was detected on chromosomes 2A and 5B. Similarly, epistatic interaction for this trait was reported by Tiwari *et al*. [32].

The QTL (*Qgfr*.*iiwbr-2A*) associated with grain filling rate under heat stress conditions on chromosomes 2A was tightly linked with marker *gwm122* and explained 8.7% PV of the trait and 12.8% of PV for HSI of the trait. Previously genomic location for grain filling rate was identified on chromosome 6A, 6B and 7D [39]. Four QTL x QTL interactions covering genomic regions on chromosomes 2A, 6D, 1A, 7A, 2D, 5B were detected for HSI of this trait which explained 1.06% to 6.62% phenotypic variance.

Identified QTLs and their co-localization with heat tolerance of yield and component traits can play significant role in heat tolerance. These traits could potentially provide underlying tolerance mechanisms and complementary selection criteria for heat stress breeding. According to Kato *et al*. [40], it’s well known that correlated traits are likely to map to similar locations. Co-location of QTLs may be due to multiple important genes in the region or due to gene(s) with pleiotropic effects. Identifying co-locations of QTLs controlling different traits will lead to markers for more effective MAS of correlated traits. In the present investigation most of the QTLs identified for yield and component traits were co-localized on chromosome 2A and were associated with either *gwm122* or *gwm448* covering 31.2cM distance. The trait associated with co-localization included TGW, GNS, GWS, GFR, GY etc. QTLs on chromosome 6D for GFR and GY under normal condition were also co-localized and associated with marker *barc196*. Co-localized QTLs have been reported in wheat for grain filling duration with grain protein content, yield and thousand grain weight [41], yield and tiller numbers [42], yield and grain numbers [43], kernel numbers and single kernel weight [37], yield components [44], senescence-related traits [45], flag leaf length and width [46], HSITGW, HSIGFD, CTD, HSIYLD [31] TGW and HSITGW [32]. Landjeva *et al*.[47] emphasized that precision of detected QTL may be increased by co-localization of QTLs in few genomic regions. During the present study, three consistent QTLs associated with GWS, GNS and TGW in most of the studied environments were present on chromosome 2A and co-localized within the *gwm448* and *wmc296* (marker interval) and showed tight linkage with marker *gwm122* rather marker embedded in the QTL region. Alleles with highest LOD scores and largest additive effects for TGW (21.01) and (GNS) -2.820 were transgressed from heat tolerant parent HD 2808. All alleles linked to maximum 30% (TGW) contribution with a significant LOD score were contributed by HD2808.

Previously reported QTLs (14) in chromosome 2A for traits under heat stress were localized in between 6-18cM upward and 59.56–150.37cM downward regions covering 12cM and 90.7cM, respectively in Somers *et al*. [24] consensus maps (Fig 2). No QTLs for heat tolerance has been reported, which was localized in 18–59.56 cM region of chromosome 2A. During present study, 17 QTLs were detected in chromosome 2A. Fifteen of these, which were associated with grain traits under heat stress, were localized in 49–52 cM region of chromosome 2A. The nearest QTL detected in earlier studies, *QFv/Fm*.*cgb-2A* [48] followed by *QYld.www.2A* [49] were approximately 6cM, 7.56cM below the presently identified QTLs region, respectively (Fig 2).

Additionally, QTLs for physiological traits *i*.*e*. chlorophyll fluorescence and its parameters and chlorophyll content, phenological traits *i*.*e*. physiological maturity, days to heading and days to anthesis and plant height under late sown and HSI traits were also detected on chromosome 2A.The LOD score of these QTLs was<3.0 while, the phenotypic variations of these QTLs were varied from 4.66 to 22.01%. QTL for HSI of plant height and physiological maturity were located in the same genomic region where QTL for grain yield components were located. Other QTLs for physiological traits and phonological traits under late sown were located 8cM and 33.5 cM below the genomic location associated with grain traits, respectively in consensus map of Somers *et al*. [24]. The identified QTL region is, therefore, unique for heat tolerance. After validation of these consistent QTLs in this hot-spot region could be used to improve heat tolerance in wheat using MAS and MAB.

## Supporting information

S1 Table Mean, range and heat susceptibility index of various grain yield and component traits in HD 2808/ HUW 510 RIL population during 2013–14 and 2014–15 crop seasons. (DOCX)

S1 Fig Post heading daily Maximum and Minimum temperature under timely and late sown conditions, A: During crop season 2013–14 B: During crop season 2014–15. (TIF)

S2 Fig Frequency distribution for HSI of various traits in HD 2808/ HUW 510 RIL population during crop seasons 2013–14 and 2014–15. (TIF)

S3 Fig Consistent QTLs identified GWS on chromosome no 2A. (TIF)

S4 Fig Consistent QTLs identified for GNS on chromosome no 2A. (TIF)

S5 Fig Consistent QTLs identified for TGW on chromosome no 2A. (TIF)

## Abbreviations

HSI: Heat susceptibility index
TS: Timely sown
LS: Late sown
GFD: Grain filling duration
BY: Biological yield
GWS: Grain Weight /main spike
GNS: Grain Number/ spike
GFR: Grain Filling Rate
TGW: Thousand Grain Weight
GY: Grain yield
