# Supplementary material for: Mapping QTLs for grain yield components in wheat under heat stress
Source: PLoS One. 2017 Dec 19;12(12):e0189594. doi: 10.1371/journal.pone.0189594 (PMC5736223; doi:10.1371/journal.pone.0189594)
Supplement: S1 Table — (DOCX) [file pone.0189594.s001.docx]

**S1 Table.** **Mean, Range and heat susceptibility index of various grain yield and component traits in HD 2808/ HUW 510 RIL population during 2013-14 and 2014-15 crop seasons**

| **Traits** | **HD 2808** | | **HUW 510** | | **RILs Mean** | | **RILs Range** | |
| --- | --- | --- | --- | --- | --- | --- | --- | --- |
|  | **TS** | **LS** | **TS** | **LS** | **TS** | **LS** | **TS** | **LS** |
| 2013-14 | | | | | | | | |
| GFD | 45.5±0.71 | 36.5±0.71 | 44.5±2.12 | 32.5±0.71 | 42.66±2.16 | 32.91±1.88 | 37.0-50.0 | 28.0-38.0 |
| GWS | 1.49±0.07 | 1.55±0.07 | 1.97±0.02 | 1.51±0.01 | 1.83±0.39 | 1.54±0.279 | 1.04-3.24 | 0.80-2.40 |
| GNS | 38.20±0.28 | 43.20±0.28 | 51.3±4.95 | 35.90±2.12 | 45.29±6.25 | 39.29±5.89 | 29.40-67.80 | 25.80-59.80 |
| TGW | 37.25±0.35 | 42.36±0.05 | 44.12±5.11 | 39.45±5.88 | 39.89±4.75 | 38.17±3.96 | 30.09-51.00 | 30.29-50.69 |
| BY | 1.91±0.12 | 1.78±0.06 | 2.65±0.02 | 1.41±0.05 | 2.49±0.36 | 1.72±0.32 | 1.66-3.49 | 1.00-2.66 |
| GY | 0.486±0.0 | 0.474±0.01 | 0.857±0.05 | 0.486±0.0 | 0.607±0.15 | 0.464±0.11 | 0.29-1.34 | 0.22-0.85 |
| GFR | 1.06±0.017 | 1.31±0.025 | 1.92±0.022 | 1.49±0.022 | 1.42±0.356 | 1.41±0.310 | 0.68-3.03 | 0.729-2.45 |
| HSIGFD | 0.87±0.0 |  | 1.18±0.06 |  | 0.99±0.24 |  | 0.23-1.66 |  |
| HSIGWS | -0.26±0.01 |  | 1.56±0.15 |  | 0.84±1.21 |  | -1.64-3.73 |  |
| HSIGNS | -0.98±0.03 |  | 2.26±0.27 |  | 0.91±1.14 |  | -1.75-3.56 |  |
| HSITGW | -3.06±0.25 |  | 2.49±0.49 |  | 0.76±3.08 |  | -7.17-8.67 |  |
| HSIBY | 0.19±0.08 |  | 1.38±0.03 |  | 0.88±0.40 |  | -0.37-1.67 |  |
| HSIGY | 0.07±0.0 |  | 1.84±0.14 |  | 0.88±0.82 |  | -0.87-2.86 |  |
| HSIGFR | -0.51±0.04 |  | 5.59±0.52 |  | 0.30±5.14 |  | -10.22-13.41 |  |
| 2014-15 | | | | | | | | |
| GFD | 41.5±0.71 | 38.5±0.71 | 40.5±0.71 | 32.5±0.71 | 39.3±2.07 | 34.5±1.6 | 31.0-45.0 | 30.0-39.0 |
| GWS | 1.91±0.07 | 2.04±0.02 | 1.98±0.07 | 1.52±0.14 | 1.91±0.41 | 1.67±0.29 | 1.06-3.76 | 0.98-2.94 |
| GNS | 44.60±3.39 | 44.2±1.13 | 46.96±0.05 | 32.6±4.24 | 46.67±5.77 | 41.16±5.91 | 33.8-68.2 | 21.80-58.80 |
| TGW | 37.75±0.35 | 40.58±0.79 | 47.08±0.92 | 39.3±6.08 | 41.01±4.29 | 39.65±3.16 | 30.13-51.0 | 31.25-49.14 |
| BY | 1.88±0.03 | 1.92±0.09 | 1.74±0.12 | 1.46±0.04 | 1.62±0.28 | 1.37±0.27 | 0.66-2.49 | 0.66-2.32 |
| GY | 0.493±0.01 | 0.532±0.05 | 0.594±0.05 | 0.394±0.02 | 0.467±0.10 | 0.370±0.08 | 0.27-0.90 | 0.21-0.79 |
| GFR | 1.18±0.043 | 1.40±0.12 | 1.45±0.12 | 1.19±0.047 | 1.19±0.027 | 1.07±0.23 | 0.659-2.463 | 0.582-2.558 |
| HSIGFD | 0.70±0.16 |  | 1.61±0.08 |  | 0.98±0.53 |  | -1.20-2.53 |  |
| HSIGWS | -0.51±0.25 |  | 1.92±0.96 |  | 0.83±1.32 |  | -2.39-4.49 |  |
| HSIGNS | 0.07±0.43 |  | 2.61±0.92 |  | 0.92±1.20 |  | -2.18-4.20 |  |
| HSITGW | -2.25±0.39 |  | 5.02±3.52 |  | 0.74±3.21 |  | -5.09-9.92 |  |
| HSIBY | -0.17±0.34 |  | 0.92±0.44 |  | 0.91±1.00 |  | -1.36-3.77 |  |
| HSIGY | -0.42±0.41 |  | 1.71±0.10 |  | 0.97±0.81 |  | -0.98-2.90 |  |
| HSIGFR | -1.92±0.98 |  | 1.78±0.09 |  | 0.79±1.93 |  | -4.28-5.24 |  |

± indicates standard error
