# Supplementary figures and images for: Mapping QTLs for grain yield components in wheat under heat stress
Source: PLoS One. 2017 Dec 19;12(12):e0189594. doi: 10.1371/journal.pone.0189594 (PMC5736223; doi:10.1371/journal.pone.0189594)

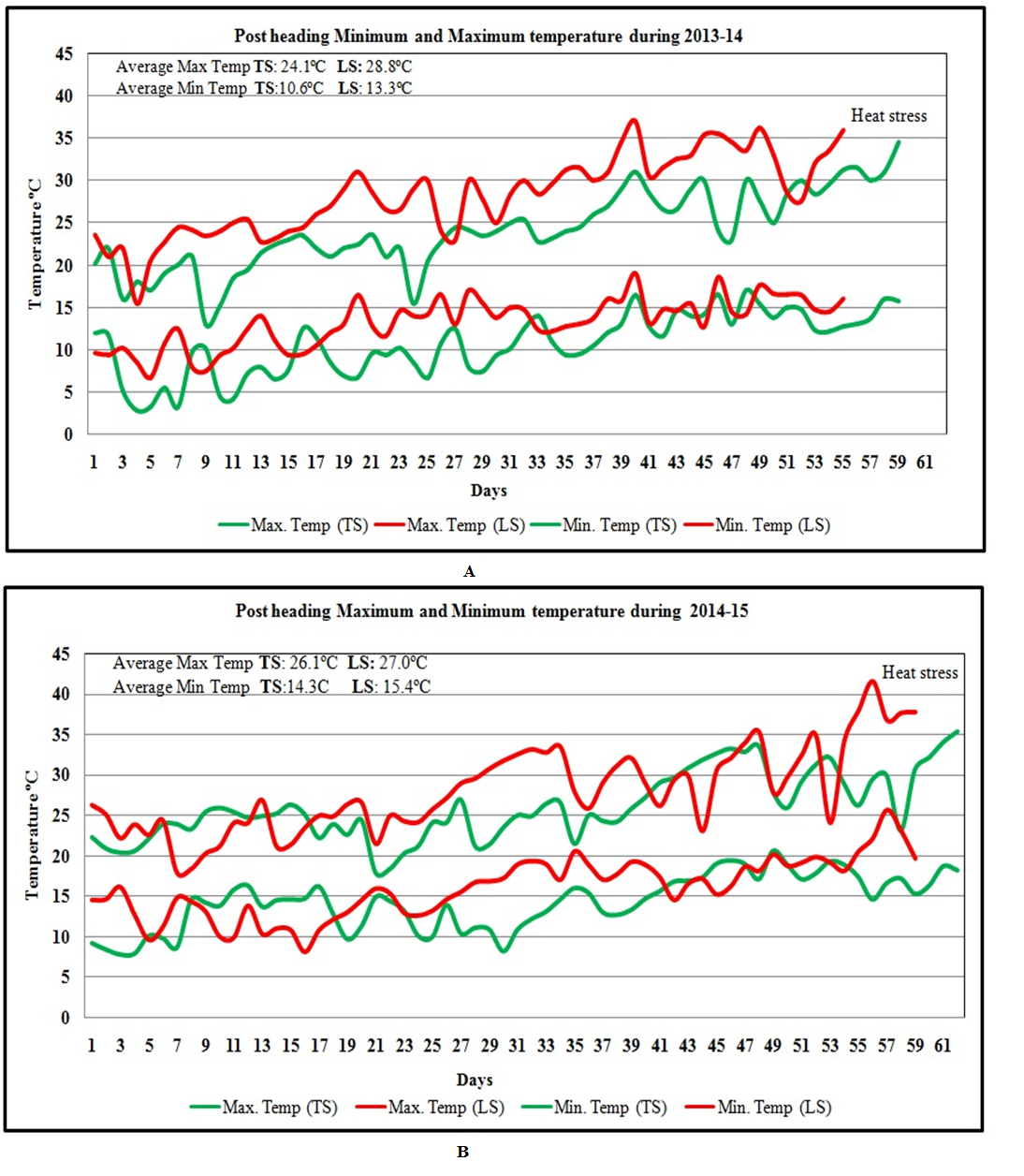

Supplement: S1 Fig — (TIF) [file pone.0189594.s002.tif]

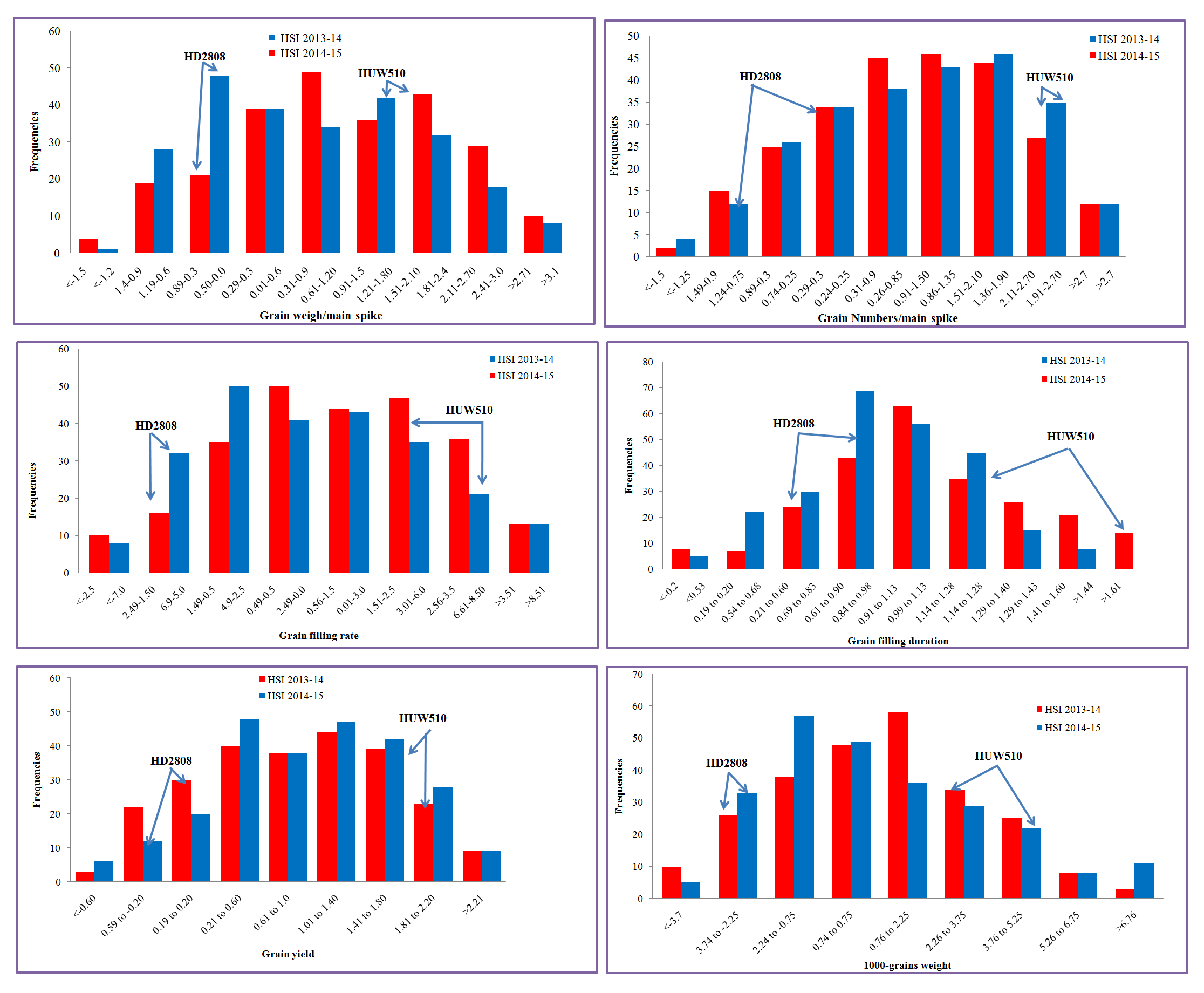

Supplement: S2 Fig — (TIF) [file pone.0189594.s003.tif]

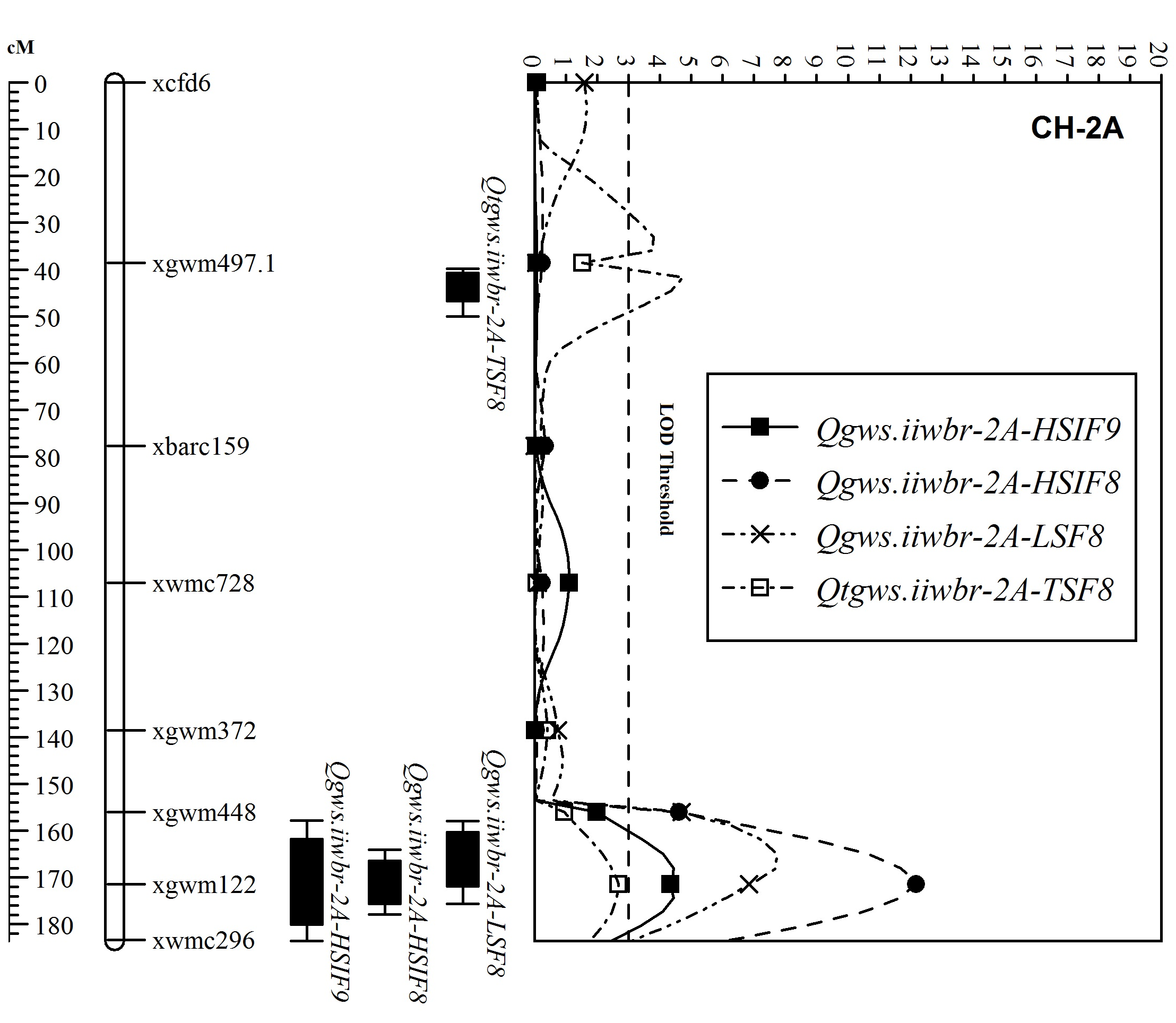

Supplement: S3 Fig — (TIF) [file pone.0189594.s004.tif]

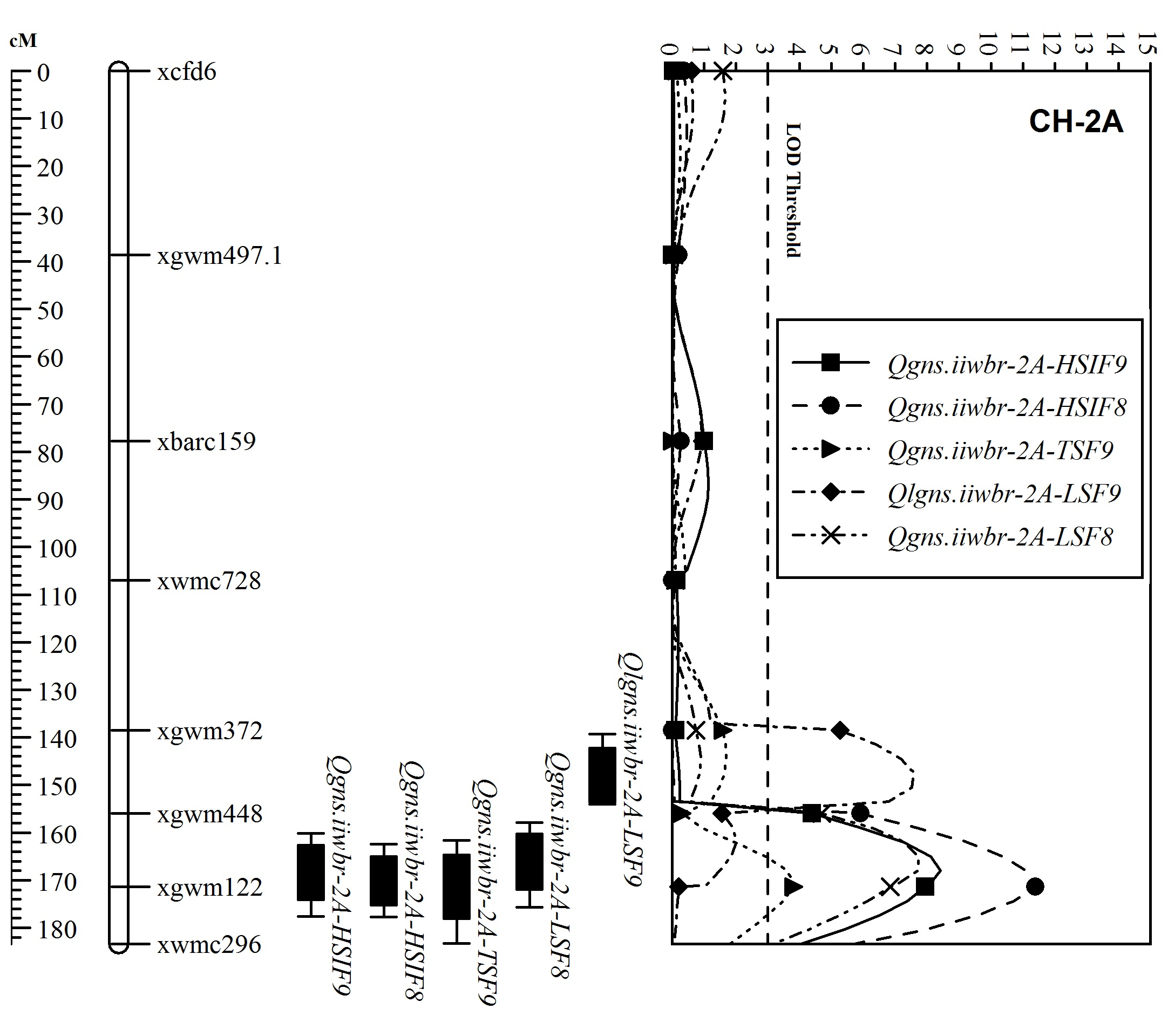

Supplement: S4 Fig — (TIF) [file pone.0189594.s005.tif]

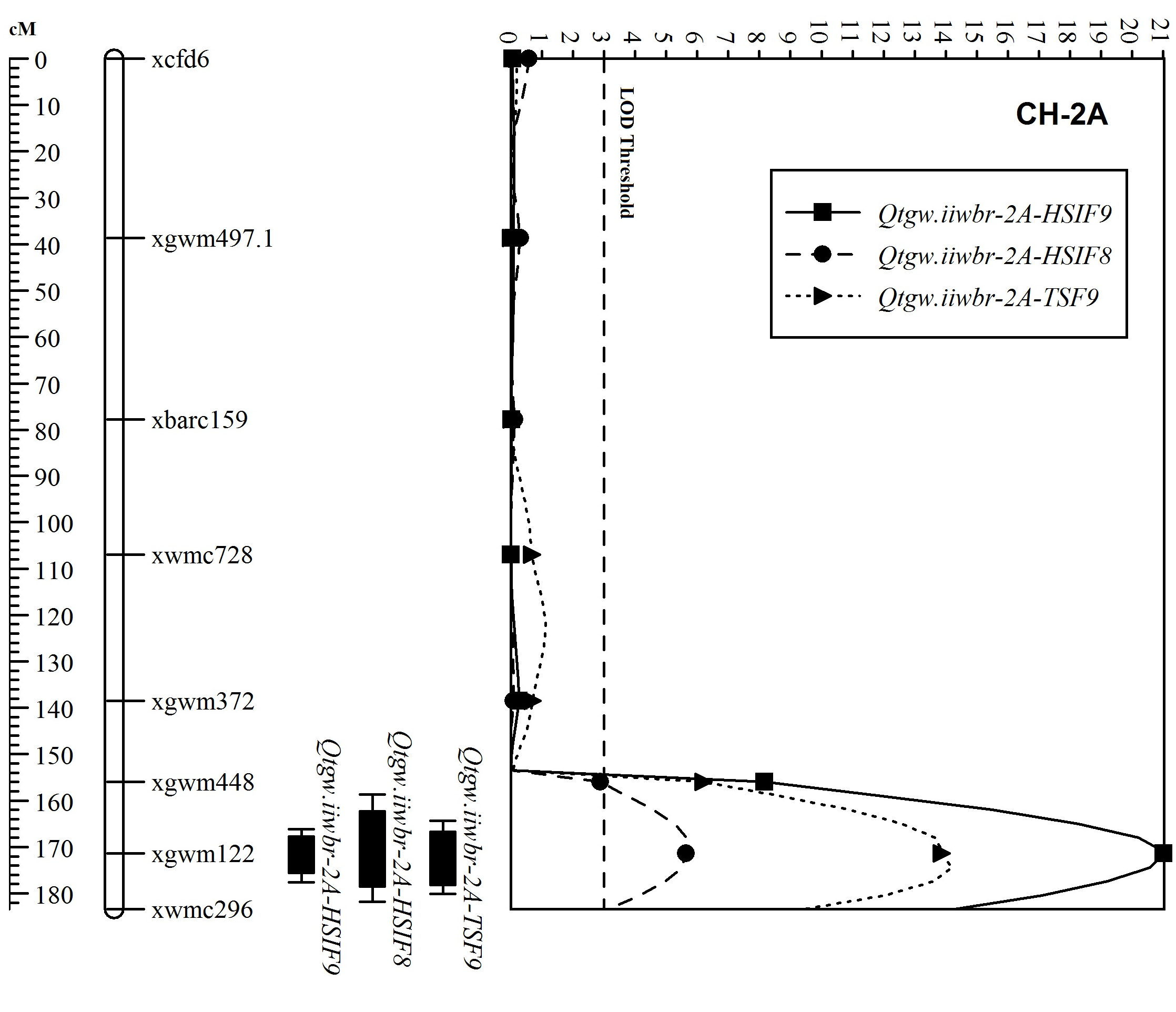

Supplement: S5 Fig — (TIF) [file pone.0189594.s006.tif]
